# Ologen augmentation of Ahmed glaucoma drainage devices in pediatric glaucomas

**DOI:** 10.1186/s12886-021-01827-4

**Published:** 2021-02-05

**Authors:** Adam Jacobson, Carin Rojas, Brenda L. Bohnsack

**Affiliations:** 1 Department of Ophthalmology and Visual Sciences, University of Michigan, 1000 Wall Street, Ann Arbor, MI 48105 USA; 2 Division of Ophthalmology, Ann & Robert H. Lurie Children’s Hospital of Chicago, 225 E. Chicago Ave, Box 70, Chicago, IL 60611 USA; 3 Department of Ophthalmology, Northwestern University Feinberg School of Medicine, 645 N. Michigan Ave, Chicago, IL 60611 USA

**Keywords:** Pediatrics, Glaucoma, Ahmed Glaucoma valve, Ologen

## Abstract

**Background:**

Limited data exists on the effectiveness of the collagen matrix, Ologen, on increasing Ahmed glaucoma valve (AGV) success in childhood glaucomas.

**Methods:**

Ocular examination and surgical details of pediatric patients who underwent AGV placement ± Ologen augmentation between 2012 and 2020. Complete success was defined as intraocular pressure (IOP) between 5 and 20 mmHg without glaucoma medications and additional IOP-lowering surgeries. Qualified success was defined as above, except IOP control maintained with or without glaucoma medications.

**Results:**

Twenty-two eyes of 16 patients underwent AGV placement of which 6 eyes had Ologen-augmentation (OAGV) and 16 eyes had conventional surgery (CAGV). Average age was 6.4 ± 5.1 years with 4.2 ± 2.5 follow-up years. There was no difference in age, number of previous surgeries, and preoperative IOP and glaucoma medications. At final follow-up, success rate was 100% (5 eyes complete, 6 eyes qualified) in the OAGV group compared to 31% (0 eyes complete, 5 eyes qualified) in the CAGV group. One and two-year survival rates were 100% for OAGV compared to 62 and 38% for CAGV. Postoperative IOP was significantly lower at 1-month and final follow-up (*p* = 0.02) as was the number of glaucoma medications at 3, 6, 12-months and final follow-up (*p* < 0.05) in the OAGV group.

**Conclusions:**

Ologen-augmentation increased the success and survival rates of AGVs in childhood glaucomas. Further, Ologen mitigated the hypertensive phase and decreased medication dependency. Longer follow-up with a greater number of eyes is required to fully evaluate the effectiveness of OAGV.

**Supplementary Information:**

The online version contains supplementary material available at 10.1186/s12886-021-01827-4.

## Background

Pediatric glaucomas are an important cause of blindness, affecting 1:5000–1:10,000 children worldwide [1]. The causes of elevated intraocular pressures (IOP) in children encompass a diverse set of pathologies including congenital abnormalities, inflammation, and trauma. Glaucoma surgery is often required to preserve vision, and the choice of surgery is dictated by numerous factors such as ocular anatomy, patient age, and end-target IOP [2].

Glaucoma drainage devices (GDDs) are a mainstay of treatment for refractory glaucoma in both pediatric and adult populations [3, 4]. In children, Ahmed glaucoma valves (AGV) have greater than 80% success rate at 1-year, but success decreases to less than 50% by 5 years [5–7]. In order to improve long-term IOP control, mitomycin C (MMC) has been used as an adjuvant during implant placement. While MMC is commonplace in trabeculectomy, its use with GDDs is controversial due to increased complications such as bleb leaks and endothelial cell damage [8]. More recently, Ologen, a biodegradable Type-I collagen matrix, has been used in glaucoma surgeries. Ologen is safe and effective in enhancing trabeculectomy surgery in adults and children [9–12]. Reports in adult patients have shown that Ologen may increase AGV success and decrease hypertensive phase incidence [13, 14]. In the current study, we compare success and survival of Ologen augmentation of AGV (OAGV) to conventional AGV (CAGV) implantation in children.

## Methods

A retrospective case series identified patients 18 years of age or younger who underwent Ahmed FP7 or FP8 (New World Medical, Rancho Cucamonga, CA) implantation at the University of Michigan between January 2012 and January 2020. This study was approved by the Institutional Review Board at the University of Michigan. Data collection was de-identified and HIPAA compliant.

Childhood glaucomas were classified based on the World Glaucoma Association consensus [15]. Glaucoma was defined by at least 2 repeated IOP measurements greater than 21 mmHg and accompanying signs of buphthalmos, corneal edema, Haabs striae, or optic nerve cupping. The primary outcome measure was AGV success. Complete success was defined as IOP of 5-20 mmHg without topical glaucoma medications and no additional IOP-lowering surgery or visually-devastating complications. Qualified success was defined as above, except IOP control maintained with or without glaucoma medications.

Data collected included age, gender, ethnicity, ocular diagnoses, intraocular surgeries, surgical procedure details, and complications. Preoperative best-corrected visual acuity (BCVA), IOP and ocular medications were last recorded values before surgery. Postoperative BCVA, IOP and ocular medications at 1-day, 1-week, 1-month, 3-months, 6-months, 12-months, and at final follow-up were recorded for secondary outcome analysis. IOP was measured by Icare (Revenio, Vantaa, Finland), Tono-pen (Reichert, Depew, NY) or Goldmann applanation.

**Fig. 1:**
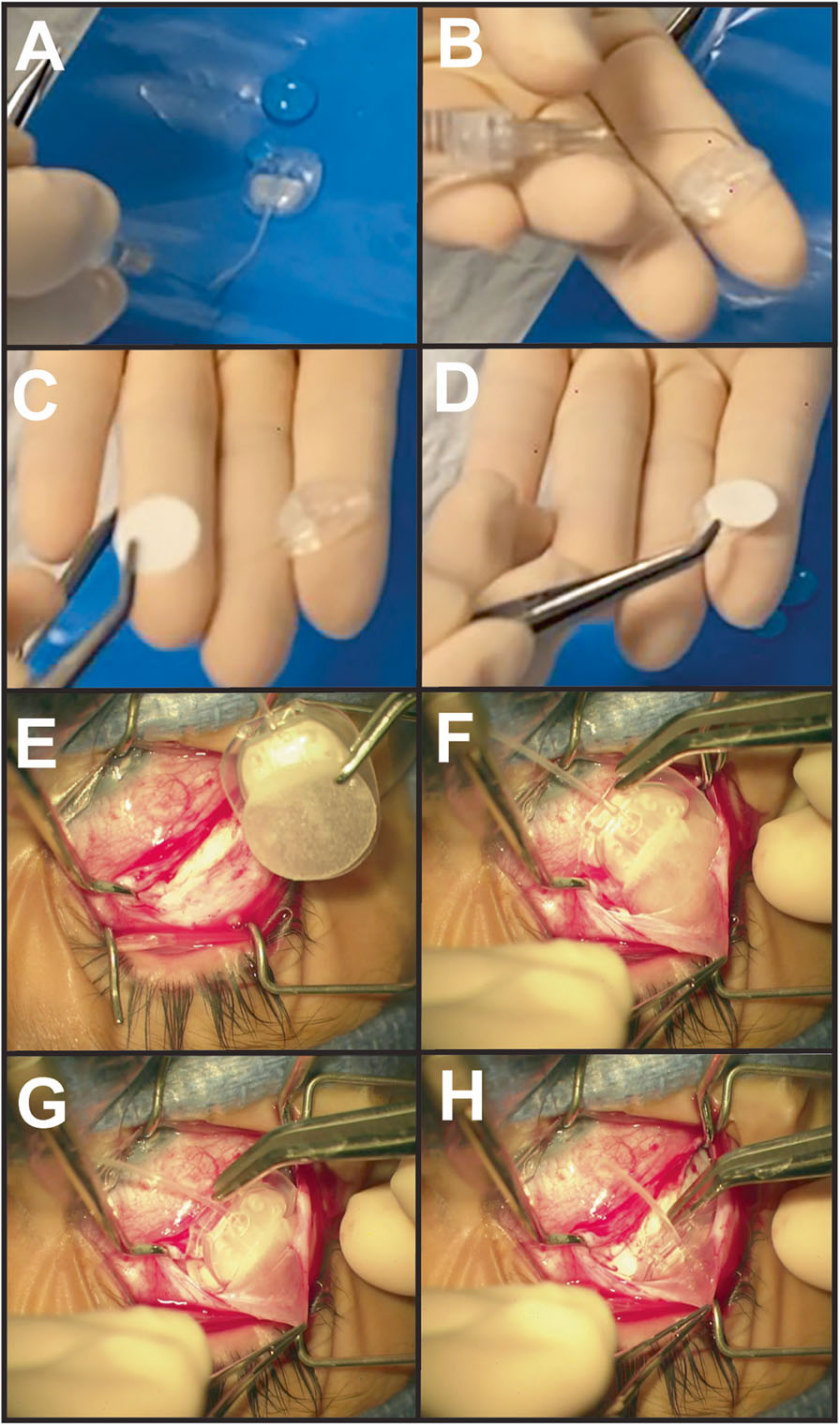
Surgical Details of OAGV Implantation. **a** The AGV was primed with balanced salt solution with a 27-gauge cannula. **b** A drop of balanced salt solution is placed on the plate **c** The 12 mm × 1 mm Ologen disc was placed on the AGV plate. **d, e** The balanced salt solution lightly hydrated the Ologen such that the disc adhered to the plate. **f, g, and h** The AGV with Ologen was placed within the sub-Tenon’s space. Care was taken to not dislodge the disc from the plate


All AGV surgeries were performed under general anesthesia by the same surgeon (BLB). The choice of AGV (FP7 vs. FP8) and location (superotemporally vs. superonasally) depended on the eye size, patient age, and history of previous ocular surgeries. The valve mechanism of the AGV was primed with balanced salt solution. For Ologen (Aeon Astron, Leiden, Netherlands) augmentation, a small drop of balanced salt solution followed by the 12 mm × 1 mm circular disc were placed on the AGV plate. The balanced salt solution lightly hydrated the Ologen such that the disc adhered to the plate (Fig. 1a-d, Video 1) Limbal-based conjunctival and Tenon’s capsule incisions were created approximately 7-8 mm from the limbus. Adjacent rectus muscles were isolated on hooks. The prepared AGV ± Ologen was placed in the selected quadrant and secured to the sclera with 8–0 nylon sutures. Care was taken in the OAGV group that the Ologen disc was not dislodged during placement (Fig. 1e-h). Dissection was carried forward to the limbus, and the tube was cut to an appropriate length. A paracentesis was created and viscoelastic was injected to deepen the anterior chamber. A 23-gauge needle was used to tunnel through the sclera, approximately 1 mm posterior to the limbus into the anterior chamber. The tube was placed through the sclerostomy and then secured to the sclera with a 9–0 nylon suture. A scleral patch graft was secured with 8–0 polyglactin sutures. Tenon’s capsule followed by conjunctiva were closed in a double-layered fashion with running 8–0 polyglactin sutures. With removal of the viscoelastic with balanced salt solution, a bleb formed over the AGV plate.

Additional file 1 Video 1: Surgical Procedure of OAGV Implantation

For statistical analyses all tests including Wilcoxon Rank Sum test (comparison between the two groups and pre- and post-operative values) and Kaplan-Meier survival curves with Log-rank (Mantel-Cox) test and accompanying 95% confidence intervals (CI) were performed with GraphPad Prism 8.0 (GraphPad, La Jolla, CA). All tests were 2-sided, and *p*-values less than 0.05 were considered statistically significant.

## Results

Twenty-two eyes of 17 patients underwent AGV implantation at 6.4 ± 5.1 years of age (range 0.4–14.1 years) with follow-up of 4.2 ± 2.5 years (Table 1). Both eyes of the 5 patients who underwent bilateral AGV implantation were included in the analyses as there was less than 1-month (expected time before onset of hypertensive phase) between surgeries. Diagnoses included primary congenital glaucoma, Axenfeld-Rieger syndrome, microphthalmia with glaucoma following cataract surgery (GFCS), uveitis, Peters Anomaly, aniridia, angle recession glaucoma, and congenital ectropion uvea (Table 2). Fifteen eyes of 11 patients were phakic, 5 eyes of 5 patients were aphakic, and 2 eyes of 1 patient were pseudophakic.

**Table 1:** Demographics

	OAGV 6 Eyes of 4 Patients	CAGV 16 Eyes of 13 Patients	
**Age (Years)**	4.6 ± 4.7	7.0 ± 5.2	p = 0.3
**Final Follow-Up (Years)**	1.5 ± 0.6	5.3 ± 2.1	p < 0.001
**Gender (M:F)**	1:5 eyes (1:3 patients)	7:9 eyes (7:6 patients)	
**Preoperative Axial Length**	18.9 ± 0.4 mm	21.2 ± 2.8 mm	p > 0.1
**Previous Intraocular Surgeries**	3 eyes of 2 patients 2.3 ± 0.6 surgeries/eye	10 eyes of 11 patients 3.3 ± 1.8 surgeries/eye	*p* = 0.4
Previous Glaucoma Surgeries	3 eyes of 2 patients 1 surgery/eye	10 eyes of 11 patients 2.6 ± 1.5 surgeries/eye	*p* = 0.1
**Lens (Phakic:Aphakic:Pseudo)**	3:1:2	12:4:0	
**Ahmed® (FP7:FP8)**	5:1	14:2	
**Plate (Supertemporal:Superonasal)**	6:0	9:7

**Table 2:** Individual Patients

CAGV	Eye	Diagnosis	Previous Intraocular Surgeries	Ahmed Type and Placement	Success/Additional Surgeries/Bleb Morphology
**Patient 1**	OD	Uveitis	CE-IOL, Synechiolysis, 180° Goniotomy	FP7 Superotemporal	Qualified High, Moderately Encapsulated Bleb
**Patient 1**	OS	Uveitis	CE-IOL, 180° Goniotomy	FP7 Superotemporal	Complete High, Moderately Encapsulated Bleb
**Patient 2**	OD	Axenfeld-Rieger	None	FP8 Superotemporal	Complete Diffuse, Low Bleb
**Patient 2**	OS	Axenfeld-Rieger	None	FP8 Superotemporal	Complete Diffuse, Low Bleb
**Patient 3**	OD	Angle Recession	None	FP7 Superotemporal	Complete High, Moderately Encapsulated Bleb
**Patient 4**	OD	Microphthalmia/GFCS	CE, Transcleral Cyclophotocoagulation	FP7 Superotemporal	Complete Diffuse, Low Bleb
**OAGV**	**Eye**	**Diagnosis**	**Previous Intraocular Surgeries**	**Ahmed Type and Placement**	**Success/Additional Surgeries/Bleb Morphology**
**Patient 5**	OD	Primary Congenital Glaucoma	Trabeculotomy, Trabeculectomy with MMC, Bleb Needling	FP7 Superonasal	Failed Baerveldt 350 Placed
**Patient 5**	OS	Primary Congenital Glaucoma	Trabeculotomy, Trabeculectomy with MMC, Bleb Needling	FP7 Superonasal	Qualified High Moderately Encapsulated Bleb
**Patient 6**	OD	Axenfeld-Rieger	None	FP7 Superotemporal	Failed Ahmed Removed and Baerveldt 350 Placed
**Patient 6**	OS	Axenfeld-Rieger	None	FP7 Superotemporal	Failed Ahmed Removed and Baerveldt 350 Placed
**Patient 7**	OD	Aniridia	Endoscopic Cyclophotocoagulation	FP7 Superotemporal	Qualified High, Moderately Encapsulated Bleb
**Patient 7**	OS	Aniridia	Endoscopic Cyclophotocoagulation	FP7 Superotemporal	Qualified High, Moderately Encapsulated Bleb
**Patient 8**	OS	Primary Congenital Glaucoma	Trabeculotomy × 2, Baerveldt 250, Tube Revision	FP7 Superonasal	Failed Transcleral Cyclophotocoagulation
**Patient 9**	OD	Microphthalmia/GFCS	CE, Transcleral Cyclophotocoagulation × 4, Baerveldt 350, Removal of Baerveldt	FP8 Superotemporal	Failed Hypotony and Ahmed Removed
**Patient 10**	OD	Microphthalmia/GFCS	CE, Pupilloplasty, Vitrectomy, Baerveldt 350	FP7 Superonasal	Qualified Diffuse, Low Bleb
**Patient 11**	OS	Axenfeld-Rieger	None	FP7 Superotemporal	Failed Ahmed Removed and Baerveldt 350 Placed
**Patient 12**	OD	MicrophthalmiaGFCS	CE, Transcleral Cyclophotocoagulation × 4	FP7 Superotemporal	Qualified Diffuse, Low Bleb
**Patient 13**	OS	Uveitis	None	FP7 Superotemporal	Failed Transcleral Cyclophotocoagulation
**Patient 14**	OS	Congenital Ectropion Uvea	None	FP7 Superotemporal	Failed Ahmed Removed, Trabeculectomy with MMC
**Patient 15**	OS	Peters Anomaly	None	FP7 Superonasal	Failed Baerveldt 350 Placed
**Patient 16**	OD	Primary Congenital Glaucoma	Trabeculotomy × 2, Trabeculectomy with MMC, CE, Vitrectomy	FP7 Superonasal	Failed Baerveldt 350 Placed
**Patient 17**	OD	Peters Anomaly	Trabeculotomy	FP8 Superonasal	Failed Transcleral Cyclophotocoagulation

Six eyes of 4 patients underwent OAGV while 16 eyes of 13 patients had CAGV (Tables 1, 2). There was no difference in age at time of surgery (*p* = 0.3), number of prior intraocular surgeries (*p* = 0.5), and preoperative axial lengths (*p* > 0.1), Details of prior intraocular surgeries are provided in Table 2. Furthermore, there was no difference in preoperative IOP (*p* = 0.9) and the number of preoperative glaucoma medications (p = 0.5) (Tables 3 and 4), There was a significant difference (*p* < 0.001) in follow-up time between the groups.

**Table 3:** IOP Measurements

	OAGV		CAGV		
	IOP in mmHg (# of Eyes)^**a**^	***P*** value Pre vs. Post	IOP in mmHg (# of Eyes)^**a**^	P value Pre vs. Post	P value OAGV vs. CAGV
**Preoperative**	30.5 ± 7.4 (6 Eyes)		29.8 ± 13.7 (16 Eyes)		p = 0.9
Postoperative					
**1 Day**	13.4 ± 9.7 (6 Eyes)	*p* = 0.009	10.9 ± 4.8 (16 Eyes)	*p* = 0.0003	p = 0.4
**1 Month**	10.5 ± 3.1 (6 Eyes)	*p* = 0.0001	17.0 ± 5.7 (15 Eyes)	*p* = 0.003	**p = 0.02**
**3 Months**	10.6 ± 5.9 (6 Eyes)	*p* = 0.0009	15.4 ± 3.8 (13 Eyes)	*p* = 0.002	*p* = 0.06
**6 Months**	11.6 ± 3.7 (6 Eyes)	*p* = 0.0006	16.7 ± 8.3 (10 Eyes)	p = 0.04	*p* = 0.2
**12 Months**	13.4 ± 4.2 (6 Eyes)	*p* = 0.001	15.0 ± 2.2 (9 Eyes)	*p* = 0.006	p = 0.4
**Final**	10.8 ± 4.1 (6 Eyes)	*p* = 0.0002	17.4 ± 3.6 (5 Eyes)	*p* = 0.05	**p = 0.02**

^a^Number of Eyes with Qualified or Complete Success

**Table 4:** Glaucoma Medications

	OAGV		CAGV		
	Medications (# of Eyes)^**a**^	P value Pre vs. Post	Medications (# of Eyes)^**a**^	P value Pre vs. Post	P value OAGV vs. CAGV
**Preoperative**	2.8 ± 1.5 (6 Eyes)		2.3 ± 1.2 (16 Eyes)		p = 0.5
Postoperative					
**1 Day**	0.2 ± 0.4 (6 Eyes)	p = 0.001	0.8 ± 0.8 (16 Eyes)	*p* = 0.0005	*p* = 0.09
**1 Month**	0 (6 Eyes)	*p* = 0.0008	0.5 ± 0.9 (15 Eyes)	p = 0.0001	*p* = 0.18
**3 Months**	0 (6 Eyes)	p = 0.0008	1.1 ± 1.3 (13 Eyes)	p = 0.02	**p = 0.05**
**6 Months**	0 (6 Eyes)	p = 0.0008	1.5 ± 1.4 (10 Eyes)	*p* = 0.11	**p = 0.02**
**12 Months**	0 (6 Eyes)	p = 0.0008	2.1 ± 1.4 (9 Eyes)	*p* = 0.7	**p = 0.002**
**Final**	0.2 ± 0.4 (6 Eyes)	p = 0.001	2.0 ± 1.0 (5 Eyes)	p = 0.6	**p = 0.003**

^a^Number of Eyes with Qualified or Complete Success

AGV placement was uncomplicated in all eyes and there were no intra-operative or immediate post-operative visually-devastating complications. In the OAGV group, at final follow-up, all 6 eyes (100%) were qualified successes with 5 eyes (83%) complete successes. Postoperative IOP at all time points (Table 3) were significantly lower than preoperative IOP (*p* < 0.001). Furthermore, none of the OAGV eyes showed a hypertensive phase between 1 and 2-months postoperatively. Similarly, the number of glaucoma medications at all postoperative time points (Table 4) was decreased (p < 0.001). In the CAGV group, at final follow-up, the qualified success rate was 31% (5 of 16 eyes). No eyes were complete successes. Ten eyes required additional glaucoma surgery (Table 2). One eye, which had previously undergone multiple sessions of transcleral cyclophotocoagulation became hypotonous and the Ahmed device was removed. Bleb morphology in the complete or qualified successes was similar in the OAGV (6 eyes) and CAGV (5 eyes) groups (Table 2). In CAGV eyes that remained qualified successful, postoperative IOPs (Table 2) were significantly lower than preoperative IOP (*p* < 0.05). Compared to preoperative, the number of glaucoma medications (Table 3) was significantly fewer at postoperative 1-day, 1-month, and 3-months (*p* < 0.02), but not at 6-months, 12-months, and final follow-up in eyes.

**Fig. 2:**
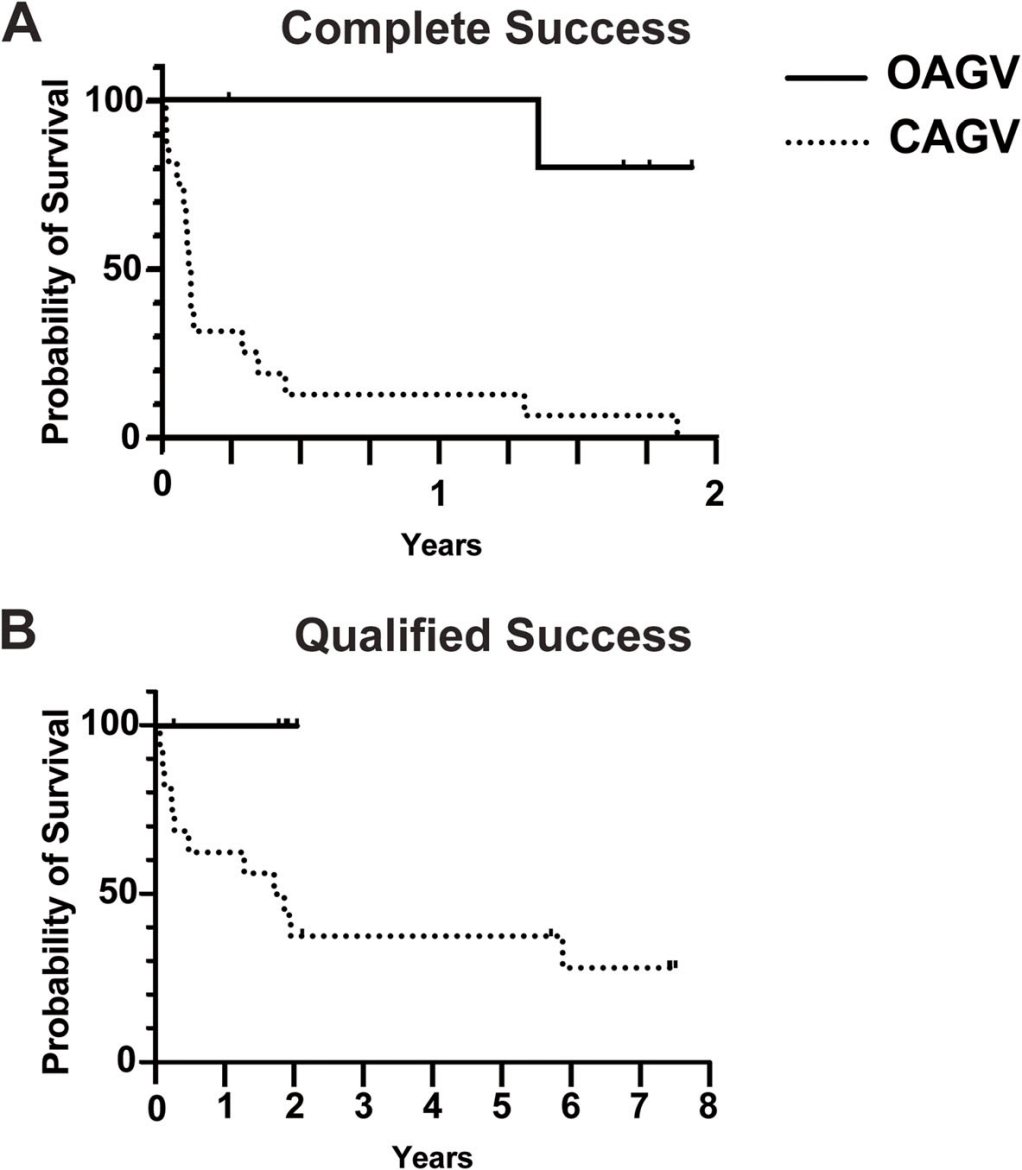
Survival Rates of OAGV and CAGV. **a** Survival curves for complete success (IOP control without glaucoma medications) of OAGV vs. CAGV were significantly different (*p* = 0.0004) by Log-rank (Mantel Cox) test. Survival rate for OAGV was 100% at 1-year and 80% with 95% CI [20, 97]. In contrast, survival rates for CAGV was 13% with 95% CI [2, 33] at 1-year and 0% at 2-years. **b** Survival curves for qualified success of OAGV vs. CAGV were also significantly different (0.04). Survival rate for OAGV was 100% at 1 and 2-years. Survival rates for CAGV was 63% with 95% CI [35, 82] at 1-year, 38% with 95% CI [16, 60] at 2-years, and 28% with 95% CI [8, 52] at 6-years

Kaplan-Meier survival analysis demonstrated significant differences between the OAGV and CAGV groups [(*p* = 0.0004 for complete success (Fig. 2a), *p* = 0.04 for qualified success (Fig. 2b)]. Two-year survival rates with OAGV for complete success was 80% with 95% CI [20, 97] and for qualified success was 100%. In contrast, 2-year survival rates with CAGV for complete success was 0% and for qualified success was 37% with 95% CI [15, 49]. Comparing OAGV eyes to CAGV eyes which had remained qualified successful, IOP at 1-month postoperatively and final follow-up was significantly lower (*p* = 0.02) in the OAGV group. Furthermore, the number of glaucoma medications was significantly lower in the OAGV eyes at 3-months, 6-months, 12-months, and at final follow-up (*p* < 0.05).

Given the nature of the patients with pediatric glaucoma, BCVA measurements were limited by age and cooperation. At time of AGV surgery, 2 patients (3 eyes) in the OAGV group and 4 patients (6 eyes) in the CAGV group were young infants and showed reaction to light. At final follow-up, 1 patient (1 eye) in the OAGV was still an infant while all patients in the CAGV group were cooperative for visual acuity testing. In patients able to undergo visual acuity testing, there was no significant difference in LogMar VA between the OAGV and the CAGV groups at time of surgery (0.5 ± 0.5 vs. 0.9 ± 0.9. *p* = 0.5) or at final follow-up (0.4 ± 0.2 vs. 0.8 ± 0.8, *p* = 0.6). Overall 3 eyes showed worse vision at final follow-up. One eye in the OAGV group had a decline in BCVA due to worsening band keratopathy. Two eyes in the CAGV group had worse visual acuity, 1 due to hypotony and 1 due to retinal detachment following placement of a Baerveldt GDD.

## Discussion

GDDs are important to the surgical management of pediatric glaucomas [3, 4, 16]. Although many forms of childhood glaucomas respond well to angle surgery, certain pathologies such as Peters Anomaly, Axenfeld-Rieger syndrome, and aniridia usually require trabecular meshwork-bypass surgery to gain long-term IOP control. Trabeculectomy with anti-fibrotic medications can successfully control IOP, however, with this surgery comes the lifelong risk of visually-devastating infections [17]. With the advent of GDDs, trabeculectomy, at least in the United States, has become a less popular option for the pediatric population.

Numerous types of GDDs have been introduced over the last 25 years and are divided into valved and non-valved implants. The valved-implants such as AGVs have the advantages of immediate IOP-lowering effect and less risk of hypotony as the valve regulates uni-directional flow through the device [16]. However, early post-operative aqueous humor is hypothesized to carry pro-inflammatory factors which lead to plate encapsulation and the subsequent hypertensive phase approximately 4–6 weeks postoperatively [18, 19]. Longitudinal studies have demonstrated that the presence of an early hypertensive phase predicts worse long term outcomes [18]. Furthermore, late failure can occur due to fibrovascular ingrowth into the valve mechanism [20]. In contrast, non-valved implants such as Baerveldt GDDs rely on fibrous capsule formation around the plate to serve as the main resistance to outflow. The tube must either be temporarily ligated or staged to allow time for capsule formation. The advantage of the non-valved GDDs is the ability to achieve lower IOP for longer periods of time, while the trade-offs are delayed pressure lowering effect and a higher risk of hypotony. Nevertheless, the plates of the non-valved GDDs can also become encapsulated resulting in failure, although this typically occurs later postoperatively compared to the valved-implants [21]. Both types of GDDs in pediatric glaucomas typically have a lower success rate compared to adult-onset glaucomas, presumably due to the magnified inflammatory and wound-healing response [3, 4, 16].

Various approaches have been taken to decrease plate encapsulation and improve long-term IOP control for both valved and non-valved GDDs. MMC augmentation of GDD implantation has predominantly fallen out of favor as studies have either shown an increase in bleb leaks and complications secondary to hypotony or no significant difference in IOP or number of glaucoma medications [22]. More recently, studies have utilized Ologen in conjunction with AGV implantation in adult glaucomas [13, 14, 23].

Ologen is a porous matrix consisting of 90% type-I collagen and 10% glycosaminoglycans that biodegrades in 6–12 months. The implant promotes non-cicatricial wound healing by binding fibroblasts and subsequently preventing organization into scar formation. The use of Ologen in glaucoma surgeries was first described 10 years ago as an adjuvant for trabeculectomy. Since then, studies in adults and children have demonstrated that trabeculectomy with Ologen has a similar success rate as MMC, but with fewer complications related to hypotony [9–11, 24].

To date, three studies have reported the use of Ologen as an adjuvant for AGVs in adults [13, 14, 23]. The first showed that increased AGV success correlated with the thickness of the Ologen disc. However, the forms of Ologen that were utilized in this study are no longer commercially available [13]. The second study demonstrated a higher rate of success with OAGV compared to CAGV, and no difference in the incidence of the hypertensive phase between the two groups. Interestingly, the second study found that after 6 months, there were difference in glaucoma medication requirement between the two groups. This late-onset requirement for glaucoma medications may be due to the biodegradation of the Ologen implant [14]. The third study, a randomized prospective clinical trial, reported no difference in incidence of hypertensive phase or rate of AGV success at 1-year follow-up [23]. Importantly, these studies excluded children, although the study by Kim et al. included two patients with a history of primary congenital glaucoma who underwent AGV implantation after the age of 18 years [14].

To the best of our knowledge, we present the first study that compares OAGV to CAGV implantation in pediatric glaucomas. In contrast to the studies in adults, we found a significant improvement in both qualified and complete success rates as well as overall survival with OAGV. In addition to fewer glaucoma medications, eyes which underwent OAGV showed lower IOP at 1-month and final follow-up compared to CAGV. Although in our study we have significantly longer follow-up in the CAGV group, survival analysis was purposely done at 1 and 2 years when there was follow-up from the majority of the OAGV eyes. This analysis demonstrated that the greatest decrease in both complete and qualified success in the CAGV group occurred within the first 2 years. This was in contrast to the OAGV eyes which showed consistent survival and success during this same time frame. While it is possible that the OAGV group may have a delayed, but similar failure rate after year 2, the Ologen disc degrades approximately 6 months post-implantation. Thus, our 1 to 2-year data in the OAGV groups suggest that the presence of the Ologen itself is not required for maintenance of surgery success. While this is encouraging, longer follow-up is required to determine whether there is late failure.

While our study confirms that Ologen is a safe adjunct to AGV placement, the main downside is the cost of the biodegradable implant which is approximately $200 per unit. The placement of the Ologen disc does not increase the length of the procedure and at least in our study, has improved success and survival rates of the AGVs. In the long-term, this decreases the post-operative costs of anti-hypertensive medications and additional glaucoma surgeries.

Another potential use for Ologen may be in revisions of encapsulated AGVs. Removal of the fibrous capsule improves short-term IOP control, but does not typically yield long-term results due to frequent re-encapsulation. Unfortunately, Ologen does not seem to improve these results in cases of adult glaucoma [25]. Anecdotally, we have also seen minimal benefit of Ologen in revision of AGVs in pediatric glaucomas.

Limitations of our study include the small number of patients, the retrospective nature of the case series, and variable times of follow-up. In addition, our study represents a diverse set of pediatric glaucoma etiologies in both the OAGV and CAGV groups. The specific types of glaucoma may influence the overall success of any pressure lowering surgery including AGV placement with or without Ologen. For example, IOP control in uveitic glaucoma is highly dependent on the peri-operative inflammation control. The OAGV patient with uveitic glaucoma (patient 1) had excellent long-term inflammatory control prior to and following AGV placement in contrast to the CAGV patient with uveitic glaucoma (patient 13), whose peri-operative inflammatory control was tenuous. However, many of the other forms of glaucoma represented in this study such as Axenfeld-Rieger syndrome, Peters Anomaly, microphthalmia, and aniridia are well-known to be refractory to both medications and angle surgery. Only a handful of studies have been able to access outcomes in these especially rare diseases [26, 27]. More common forms of pediatric glaucomas such as primary congenital glaucoma and glaucoma following cataract surgery are often amenable to angle surgery and only the more complicated and difficult cases progress to trabecular bypass surgery. Thus, larger studies are required to determine whether the type of glaucoma affects success of OAGV At this time, the authors still predominantly use Baerveldt GDDs except in cases where immediate pressure lowering effect is needed. However, with longer follow-up of these initial patients and possibly a prospective trial with a higher volume of patients, OAGV implantation may become a first-line surgical treatment in forms of glaucoma not amenable to angle surgery.

## Conclusions

In our small study, Ologen-augmentation improved the success and survival rates of AGVs in refractory pediatric glaucomas. Furthermore, Ologen eliminated the hypertensive phase often seen in conventional AGV implantation and decreased the need for postoperative IOP-lowering medications. A longer follow-up with a greater number of eyes is required to fully evaluate the effectiveness of OAGV.

### Literature search

A comprehensive literature search in PubMed was conducted using keywords: Glaucoma drainage device, Ahmed glaucoma implant/device, Ologen, collagen matrix, pediatric glaucoma.

## Data Availability

The datasets used and analyzed during the current study are available from the corresponding author on reasonable request.

## Abbreviations

AGV: Ahmed Glaucoma Valve
OAGV: Ologen-augmented Ahmed Glaucoma Valve
CAGV: Conventional Ahmed Glaucoma Valve
IOP: Intraocular pressure
GDD: Glaucoma Drainage Device
MMC: Mitomycin C
CI: Confidence Interval

## References

[1] Papadopoulos M, Cable N, Rahi J (2007). The British infantile and childhood Glaucoma (BIG) eye study. Invest Ophthalmol Vis Sci.

[2] Chang I, Caprioli J, Ou Y (2017). Surgical management of pediatric glaucoma. Dev Ophthalmol.

[3] Netland PA, Walton DS (1993). Glaucoma drainage implants in pediatric patients. Ophthalmic Surg.

[4] O'Malley Schotthoefer E, Yanovitch TL, Freedman SF (2008). Aqueous drainage device surgery in refractory pediatric glaucomas: I. Long-term outcomes. J AAPOS.

[5] Dave P, Senthil S, Choudhari N, Sekhar GC (2015). Outcomes of Ahmed valve implant following a failed initial trabeculotomy and trabeculectomy in refractory primary congenital glaucoma. Middle East Afr J Ophthalmol.

[6] Al-Haddad C, Al-Salem K, Ismail K, Noureddin B (2018). Long-term outcomes of Ahmed tube implantation in pediatric glaucoma after multiple surgeries. Int Ophthalmol.

[7] Pakravan M, Esfandiari H, Yazdani S (2019). Clinical outcomes of Ahmed glaucoma valve implantation in pediatric glaucoma. Eur J Ophthalmol.

[8] Gross FJ (1994). Six month success of Krupin valve with and without Mitomy-cin-C in the treatment of complicated glaucomas. Invest Ophthalmol Vis Sci.

[9] He M, Wang W, Zhang X, Huang W (2014). Ologen implant versus mitomycin C for trabeculectomy: a systemic review and meta-analysis. PLoS One.

[10] El-Sayyad F, El-Saied HMA, Abdelhakim MASE (2017). Trabeculectomy with ologen versus mitomycin C in juvenile open-angle glaucoma: a 1-year study. Ophthalmic Res.

[11] Singab AAS, Mohammed OA, MSaleem MIH, Abozaid MA (2017). A comparative study: the use of collagen implant versus mitomycin-C in combined trabeculotomy and trabeculectomy for treatment of primary congenital glaucoma. J Ophthalmol.

[12] Singh K, Bhattacharyya M, Mutreja A, Dangda S (2018). Trabeculectomy with subconjunctival collagen implant in Indian eyes: long-term results.

[13] Song M, Lee S, Choe D (2017). Collagen matrices for glaucoma drainage device implantation. Invest Ophthalmol Vis Sci.

[14] Kim TJ, Kang S, Jeoung JW (2018). Comparison of 1-year outcomes after Ahmed glaucoma valve implantation with and without Ologen adjuvant. BMC Ophthalmol.

[15] Weinreb RN, Grajewski AL, Papadopoulos M (2013). Childhood Glaucoma. The 9th consensus report of the world Glaucoma association.

[16] Malik R, AlDarrab A, Edward DP (2020). Conemporary management of refractory pediatric glaucoma. Curr Opin Ophthalmol.

[17] Luebke J, Neuburger M, Jordan JF (2019). Bleb-related infections and long-term follow-up after trabeculectomy. Int Ophthalmol.

[18] Nouri-Mahdavi K, Caprioli J (2003). Evaluation of the hypertensive phase after insertion of the Ahmed Glaucoma valve. Am J Ophthalmol.

[19] Jung KI, Park H, Jung Y, Park CK (2015). Serial changes in the bleb wall after glaucoma drainage implant surgery: characteristics during the hypertensive phase. Acta Ophthalmol.

[20] Tung I, Marcus I, Thiamthat W, Freedman SF (2014). Second glaucoma drainage devices in refractory pediatric glaucoma: failure by fibrovascular ingrowth. Am J Ophthalmol.

[21] Elbaklish KH, Saleh SM, Gomaa WA (2020). Baerveldt glaucoma implant versus Ahmed glaucoma implant in a one-year follow up, comparative study. Clin Ophthalmol.

[22] Costa VP, Azuara-Blanco A, Netland PA (2004). Efficacy and safety of adjunctive mitomycin C during Ahmed glaucoma valve implantation: a prospective randomized clinical trial. Ophthalmology.

[23] Sastre-Ibáñez M, Cabarga C, Canut MI (2019). Efficacy of ologen matrix implant in Ahmed glaucoma valve implantation. Sci Rep.

[24] Cillino S, Casuccio A, Di Pace F (2016). Biodegradable collagen matrix implant versus mitomycin-C in trabeculectomy: five year follow-up. BMC Ophthalmol.

[25] Salimi A, Kovalyuk N, Harasymowycz PJ (2019). Tube shunt revision with excision of fibrotic capsule using mitomycin C with and without ologen-a collagen matrix implant: a 3-year follow-up study. J Glaucoma.

[26] Dolezal KA, Besirli CG, Mian SI, Sugar A, Moroi SE, Bohnsack BL (2019). Glaucoma and cornea surgery outcomes in Peters anomaly. Am J Ophthalmol.

[27] Zepeda EM, Branham K, Moroi SE, Bohnsack BL (2020). Outcomes of glaucoma associated with Axenfeld-Rieger syndrome. BMC Ophthalmol.

